# Dense Helical Electron Bunch Generation in Near-Critical Density Plasmas with Ultrarelativistic Laser Intensities

**DOI:** 10.1038/srep15499

**Published:** 2015-10-27

**Authors:** Ronghao Hu, Bin Liu, Haiyang Lu, Meilin Zhou, Chen Lin, Zhengming Sheng, Chia-erh Chen, Xiantu He, Xueqing Yan

**Affiliations:** 1 State Key Laboratory of Nuclear Physics and Technology, and Key Laboratory of HEDP of the Ministry of Education, CAPT, Peking University, Beijing, 100871, China; 2 Institute of Applied Physics and Computational Mathematics, Beijing, 100088, China; 3 Department of Physics, Shanghai Jiao Tong University, Shanghai, 200240, China

## Abstract

The mechanism for emergence of helical electron bunch(HEB) from an ultrarelativistic circularly polarized laser pulse propagating in near-critical density(NCD) plasma is investigated. Self-consistent three-dimensional(3D) Particle-in-Cell(PIC) simulations are performed to model all aspects of the laser plasma interaction including laser pulse evolution, electron and ion motions. At a laser intensity of 10^22^ W/cm^2^, the accelerated electrons have a broadband spectrum ranging from 300 MeV to 1.3 GeV, with the charge of 22 nano-Coulombs(nC) within a solid-angle of 0.14 Sr. Based on the simulation results, a phase-space dynamics model is developed to explain the helical density structure and the broadband energy spectrum.

High energy electron beams have broad applications in research fields like high energy physics, Inertial Confined Fusion Fast Ignition scheme^1^, radiography^2^, synchrotron radiation^3^,^4^,^5^,^6^,^7^, etc. Laser plasma accelerators^8^,^9^,^10^, first proposed by T. Tajima and J. M. Dawson^11^, have draw much attension due to its huge acceleration gradients, typically on the order of tens to hundreds of GV/m. Remarkable advances have been made both theoretically and experimentally. Two significant acceleration regimes have been proposed, known as the laser wake-field acceleration(LWFA)^11^,^12^,^13^,^14^,^15^,^16^,^17^ and the direct laser acceleration(DLA)^18^,^19^.

LWFA regime has been studied thoroughly in theories^8^,^11^,^13^,^14^, simulations^12^,^14^,^15^,^20^ and experiments^16^,^17^,^21^,^22^,^23^,^24^. It is promising for generating high energy monoenergetic electron beams with the energies upto several GeVs^16^,^17^,^21^,^22^,^23^,^24^. However, dephasing, or the phase slipping between the ultrarelativistic electrons and the subluminal acceleration fields, limits the energy gain of LWFA. To achieve a longer acceleration length before dephasing^22^, low density plasmas, typically with *n*_*e*_ < 10^20^ cm^−3^, were used in LWFA. As a consequence, the total charges of accelerated bunches are limited under 10 nC^20^,^25^,^26^,^27^.

At higher plasma densities, the DLA mechanism makes a significant contribution or even become the dominant to accelerate electrons^20^,^28^. In DLA regime, only a small fraction of electrons can be accelerated to relatively high energy^2^,^18^,^19^,^29^, which strongly limits its potential applications. To obtain more energetic electrons within a single laser shot, higher laser intensity and higher plasma density should be employed. Higher laser intensity can be expected as more powerful femtosecond laser could be available in the near future^30^,^31^,^32^. Higher density plasmas, like near-critical density(NCD) plasmas, can be deployed for electron acceleration^33^.

The density modulation of electrons can be seen as a sign of DLA^18^. HEB was observed with PIC simulations in ref. 33 but the mechanism of its emergence lacks proper physical explanation. Besides, the performance of DLA with laser intensity over 10^21^ W/cm^2^ has not been studied systematically. At relatively low laser intensities, the energy spectrums of electrons were Maxwellian-like with the “effective temperature” grows as the square root of the intensity^18^,^19^. When only the DLA electrons were selected from the heated background plasma in the PIC simulations, the energy spectrum is broadband which contains more information of the DLA performance^34^. In this paper, a theoretical model of electron phase-space dynamics was presented to explain the mechanism for emergence of HEB and PIC simulations were performed in a slab of NCD plasma interacting with a circularly polarized laser pulse, which was focused to a peak intensity of 10^22^ W/cm^2^  (ref. 44).

## Simulation and Results

**Figure 1:**
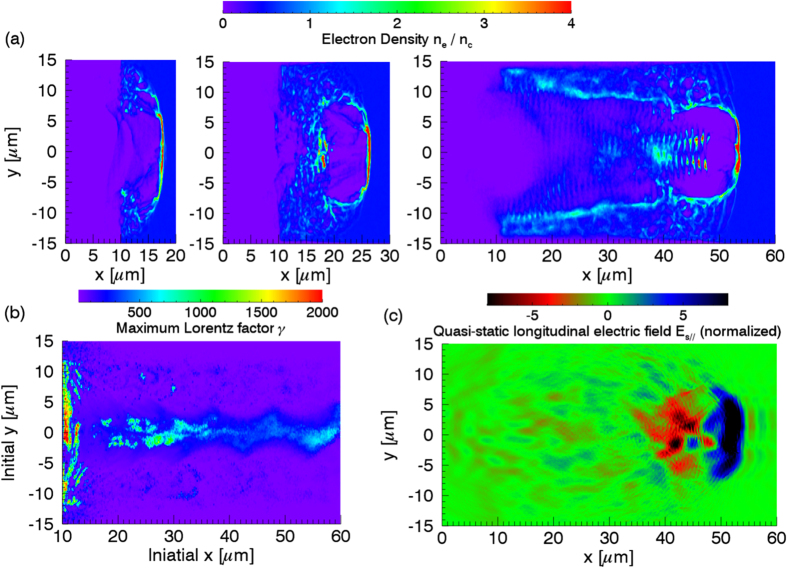
(**a**) Electron density at *t* = 20 *T*_*L*_, 30 *T*_*L*_ and 60 *T*_*L*_. (**b**) The maximum Lorentz factor *γ* for electrons from different intial positions. (**c**) The longitudinal electric field *E*_*S*‖_ at *t* = 60 *T*_*L*_.

**Figure 2:**
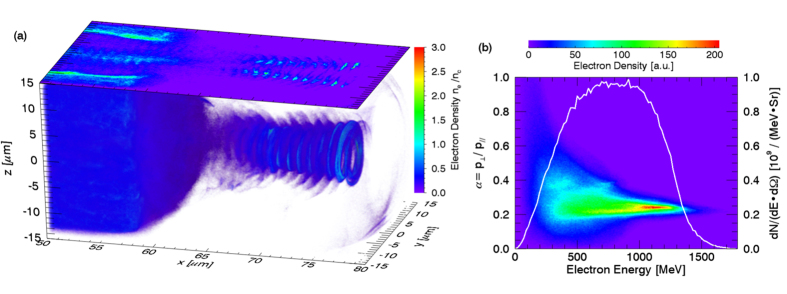
(**a**) The volume plot and contour plot on the *z* = 0 plane of the electron density at *t* = 90 *T*_*L*_. (**b**) The electon density distribution in phase-space (*γ*, *α* = *p*_?_/*p*_‖_) at the same time. The electrons are selected from the the cylindrical volume from *x* = 62 *μm* to 80 *μm*, with a radius of 6 *μm*. The white line is the energy spectrum with 0.2 < *α* < 0.3. The spectrum should be similar to that of a detector placed at an angle of 14° ± 1.7° to the laser propagation direction.

**Figure 3:**
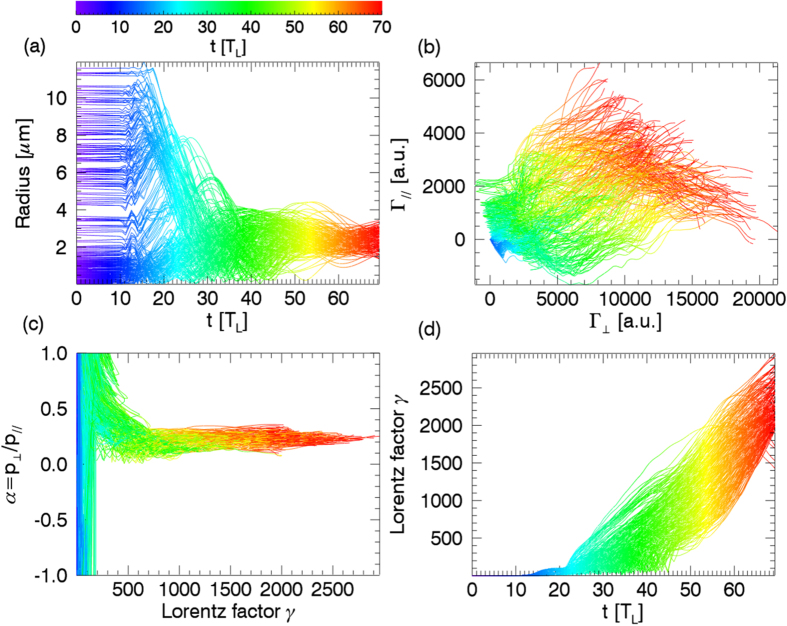
The trajectories of the PIC sample electrons in phase-space (a) (*t*, *r*), (b) (*γ*, *t*), (c) (α = *p*_?_/*p*_‖_, *γ)*, (d) (Γ_?_, Γ_‖_) from *t* = 0 *T*_*L*_ to 70 *T*_*L*_.

The PIC simulation was performed with 3D KLAP codes^35^,^36^,^37^. The circularly polarized laser pulse is incident from the left boundary of the simulation box. The simulation box is sampled by 1000 cells in light propagation direction and 240 cells in each transverse direction, corresponding to a volume of 80 *μ*m × 30 *μ*m × 30 *μ*m in real space. The incident laser pulse, with a wavelength of *λ*_*L*_ = 1.0 *μ*m, is focused to a spot diameter of 3.6 *μ*m in full width at half maximum(FWHM) with a Gaussian transverse field profile, which results in a peak intensity of about 10^22^ W/cm^2^. The focus is inside the simulation volume 40 *μ*m away from the left boundary. The laser pulse has a trapezoidal envelope in time domain, with linear rising and falling edges of 5 *T*_*L*_, where *T*_*L*_ is the light period, and a total duration of 45 *T*_*L*_ in FWHM. The target is located between 10 *μ*m to 60 *μ*m from the left boundary, with an uniform electron density of 5.5 × 10^20^ cm^−3^, about half of the critical density for light with a wavelength of 1 *μ*m. Two species of particles are included in the simulations, electrons and protons, with about 2.88 × 10^8^ macro-particles for each specie. The simulation can be divided into injection and acceleration stages. The injection stage is before 30 *T*_*L*_. A bubble-like structure was formed near the target surface, shown in Fig. 1(a), after the pulse front hits the target surface. Electrons were expeled from light axis by the transverse ponderomotive force, but the ions were barely moved, thus the transverse quasistatic electric field was formed due to the charge separation. The injection process, similar to the self-injection process of the LWFA^12^,^13^,^38^,^39^, is not continuous as most of the accelerated electrons are from the front surface of the target, shown in Fig. 1(b). An over-critical density electron bunch was formed at the rear of the bubble, shown in Fig. 1(a), and the rest of electrons were prevented from injection by the strong radial electric field generated by the bunch. The bubble-like structure evolves into a plasma channel in the following acceleration stage. The electrons gain energy through the strong light field and longitudinal quasi-static electric field inside the channel, shown in Fig. 1(c). Along with the acceleration, the electron bunch is modulated to a helix by the light field with the thread pitch roughly equal to the light wavelength, as shown in Fig. 2(a). After exiting the target, the helical electron bunch will spread with a cone angle of 14° ± 1.7°, shown in Fig. 2(b). The output energy spectrum, which is broadband and roughly from 300 MeV to 1.3 GeV, is very different from the Maxwellian-like spectrums of previous work of DLA^2^,^18^,^19^,^29^. Particle movement tracking were performed to have an insight view of the behavior of the high energy electrons in the acceleration stage. It could be found that the radii of the electrons are varying slowly in the acceleration stage and finally close to each other when exit from the acceleration stage even they are initially different as shown in Fig. 3(a). The trajectories of electrons in phase-space (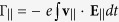, 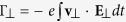) (‖ indicates the direction of light propagation, ? indicates the directions perpendicular to the light propagation, **v** is the velocity of electron and **E** is the electric field), shown in Fig. 3(b), indicate that electrons gain energies in the longitudinal and the transverse directions, but the major part is in the transverse directions, which indicates that DLA is dominant mechanism during the acceleration^19^,^29^. Figure 3(c) shows that the ratios of momentums of the transverse against the longitudinal are almost contants in the acceleration stage, which is coincident with Fig. 2(b). Figure 3(d) shows that the energies of electrons increase almost linearly with time and the maximum acceleration gradient is over 20 TeV/m.

## Electron Phase-space Dynamics

The behavior of electrons in the phase-space (*ψ*, *γ*) is the key to explain the HEB generation and acceleration mechanism. Here *ψ* is the ponderomotive phase and *γ* is the Lorentz factor of electrons. *ψ* can be seen as the relative angle of the light electric field vector and the electron transverse momentum, i.e. *ψ* = *θ* − *ϕ*, where *θ* and *ϕ* are the polar angles of the electron transverse momentum and light electric vector respectively. *γ* is the Lorentz factor of electrons. Electron motions inside the ion channel are already well-understood from the previous work^15^,^18^,^29^,^33^. Electrons inside the ion channel are trapped by the strong self-generated quasi-static electric and magnetic fields^18^,^33^,^40^, i. e., the radial electric field **E**_*Sr*_, the azimuthal magnetic field **B**_*Sθ*_ and the longitudinal magnetic field **B**_*Sz*_. The trapped electrons undergo betatron-like oscillations in the self-generated fields, the transverse Lorentz equation can be written as


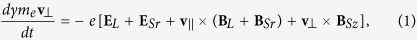


where *m*_*e*_ is the electron mass at rest, **E**_*L*_ and **B**_*L*_ are the light electromagnetic fields. In the SI units, *E*_*L*_ = *v*_*ph*_ · *B*_*L*_, where *v*_*ph*_ is the phase velocity of light in plasma and is larger than the light velocity in vacuum *c*^18^. As the electrons co-propagate with the laser pulse, the **v**_‖_ × **B**_*L*_ force is antiparallel to the electric force **E**_*L*_ and the total Lorentz force of the light field, **F**_*L*_ = −*e*(**E**_*L*_ + **v**_*L*_ × **B**_*L*_), is small compared to the Lorentz force of the quasi-static fields. The PIC simulation results show that the term **v**_?_ × **B**_*Sz*_ can be neglected comparing to other terms. Based on simulations and previous work^18^,^33^,^40^, the radial electric field and the azimuthal magnetic field profile near the light propagation axis can be written as 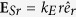, 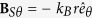, where *k*_*E*_ and *k*_*B*_ are constants, 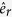 and 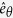 are the unit vectors of the radial and azimuthal directions respectively. Although the projected transverse motion is elliptical, as shown in Fig. 3(a), one can always use a simple circular motion to approximate. Assuming the electrons undergo circular motions with fixed radii, the angular frequency of the circular motion, i.e. the betatron frequency then can be derived from Eq. (1) as^18^,^29^,^41^


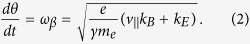


The light frequency witnessed by the electrons is 
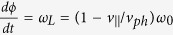
, where *ω*_0_ is the light frequency in laboratory frame. The time derivative of the ponderomotive phase can be written as


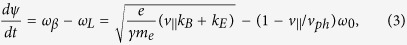


The time derivative of the electron energy is


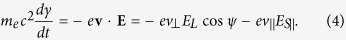


Here *E*_*Sr*_ is ignored as the radii of electrons vary slowly in the acceleration stage as shown in Fig. 3(a). Also according to PIC simulations, in the acceleration stage, *v*_?_, *v*_‖_, *k*_*B*_, *k*_*E*_, and *E*_*S*‖_ can be treated as constants as all of them vary slowly enough. A Hamiltonian can be obtained from Eqs. (3) and (4) as the phase-space spanned by (*ψ*, *γ*) is conserved.


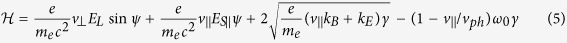


**Figure 4:**
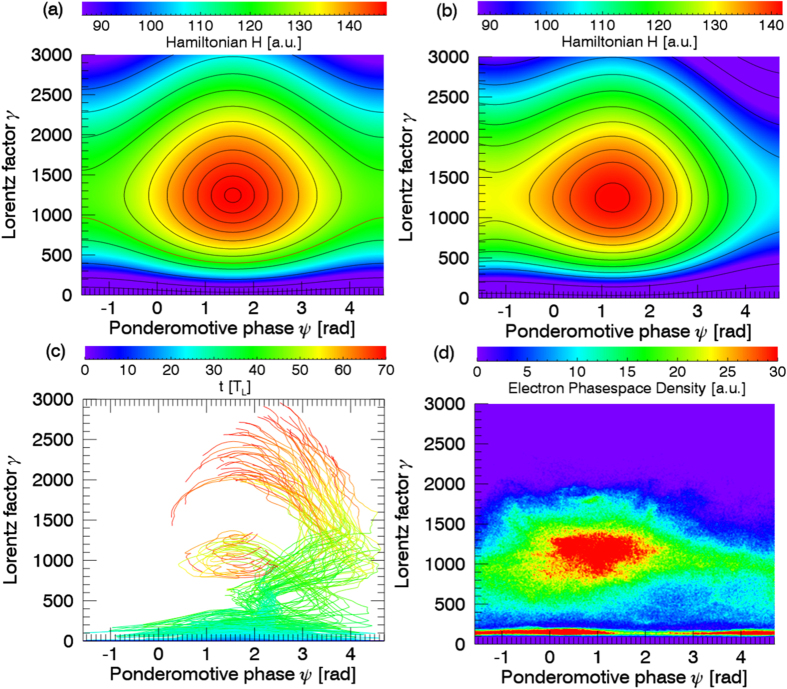
(**a**) Hamiltonian and tr**a**jectories in phase-space (*ψ*, *γ*), with longitudinal electric field ignored. (**b**) Same as (**a**), but with longitudin**a**l electric field included. Although the phase-space is not periodic, the trajectries are mannually arranged into one period as *ψ* is redefined as the remainder 
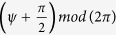
. (**c**) Trajectories of the PIC sample electrons. (**d**) Electron density distribution in phase-space, selected from the volume from *x* = 44 *μm* to 49 *μm*, within the radius of 5 *μm* at *t* = 60 *T*_*L*_.

For convenience, *ψ* is redefined as the remainder of *ψ* divided by 2*π*, noted as 
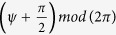
 in the following discussion. The ponderomotive phase indicates the direction of energy exchange between electrons and the light field. The electrons decelerate when 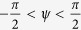, and accelerate when 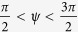. When the longitudinal electric field is ignored in Eq. (5), the Hamiltonian 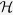 is symmetrical and periodic, and there is a separatrix and a fixed point at phase 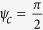 and Lorentz factor 
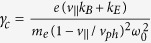
 in the phase-space, as shown in Fig. 4(a). The orbits of electrons, initially located between the separatrix and the fixed point, are closed and the ponderomotive phases of these electrons grow and then reduce slowly, so they can stay in the acceleration phase longer enough and get effectively accelerated. The electrons below the separatrix can never be accelerated because their orbits are open and their phases grow so rapidly that they can not stay in the acceleration phase long enough. With the longitudinal electric field included, the electrons below the separatrix are first accelerated by the longitudinal field, and when they are above the separatrix, they experience slower phase movements and gain energy in the acceleration phase, shown in Figs. 3(d), 4(b) and 4(c). In the theoretical model, the fixed point mentioned above is a center node, which means electrons can not get close to it. But in PIC simulations, some electrons move around the center similar as in the theoretical model while some others get close to this fixed point and are trapped nearby, causing the electron density around this fixed point to be larger than that elsewhere, as shown in Figs. 4(c) and 4(d). This is because the parameters, which are assumed to be constants in the theoretical model, may be varying slowly in time and space, which leads to the crossing of phase trajectories and converting of the fixed point property. Moreover, the special phase-space distribution, shown in Fig. 4(d), is revealed by the helical density structure of the electron beam in real space, shown in Fig. 1(a). As most of electrons are located near the fixed point, their ponderomotive phases and radii are close to each other. The electric vector of circularly polarized light is rotating in the propagation direction, so the electrons, with similar relative polar angles to the electric vector and similar radii, form a helix with the thread pitch roughly equal to the light wavelength. Figure 4(d) also indicates that the central energy of accelerated electron beam is given by the fixed point, which is roughly *γ*_*c*_*m*_*e*_*c*^2^. The constants *k*_*E*_ and *k*_*B*_ is relative to the plasma density and can be expressed as 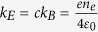
39, where *ε*_0_ is the vacuum permittivity. The phase velocity of circularly polarized long duration laser pulse can be approximated as 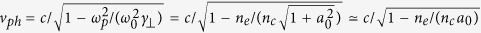
42, where *ω*_*p*_ is the plasma frequency, 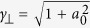 is the average transverse Lorentz factor for circularly polarized laser, *n*_*c*_ is the critical density, *a*_0_ is the normalized laser amplitude. *γ*_*c*_ can be expressed as


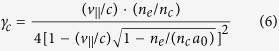


From Eq. (6), with the increase of laser intensity and plasma density, the corresponding energy of the fixed point is increasing. In the previous work^18^,^29^, the laser intensity and plasma density are small, the low energy fixed point is covered by the Maxwellian-like spectrum of the heated plasma. With the increasing of the plasma density and laser intensity, *γ*_*c*_ is also increasing and the broadband spectrum like in Fig. 2(b) can be observed.

**Figure 5:**
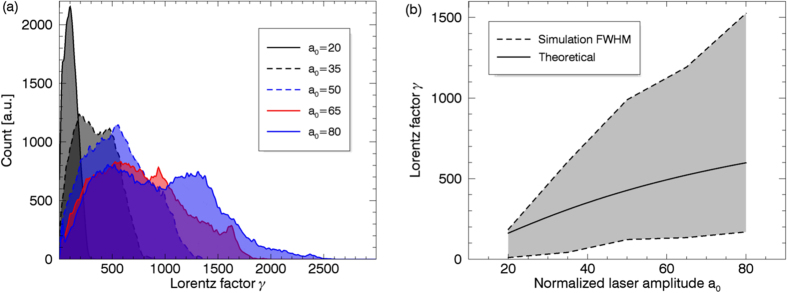
(**a**) Energy spectrums of DLA electrons with different laser amplitudes. (**b**) The FWHM of the energy spectrums in (**a**) and the Lorentz factor of the theoretical fixed point obtained with Eq. (6).

To verify the acceleration mechanism, a set of 2D PIC simulations were performed with linearly polarized laser pulses. The results are in agreement with the circularly polarized case except the electron bunches are planar rather than helical. The spectrums for different laser amplitudes, shown in Fig. 5(a), are also broadband and the bandwidths grow almost linearly with the increasing of the laser amplitudes as shown in Fig. 5(b). More energetic electrons will be obtained with higher laser intensities.

## Discussion

In this paper the mechanism for emergence of HEB from circularly polarized laser pulse propagating in NCD plasma is explained with the electron phase-space dynamics model. At a laser intensity of 10^22^ W/cm^2^, the generated HEB has a broadband spectrum ranging from 300 MeV to 1.3 GeV with a charge of 21.6 nC. With the increase of laser peak intensity, the bandwidths of the energy spectrum would grow and more energetic electrons would be obtained. Electron bunches with helical density structures are promising for generating synchrotron radiations with angular momentums^43^.

## Methods

The 2D PIC simulations are performed with fixed plasma density of 0.5 *n*_*c*_, and target length of 60 *μ*m. The laser pulses have the same duration of about 300 femtoseconds and waist radius of 3 *μ*m. The energy spectrums are for electrons selected from the area from 10 *μ*m to 20 *μ*m behind the target within a radius of 5 *μ*m and a spreading angle of ±25°.

Figures 4(a,b) are obtained with the parameters as follows: *E*_*L*_ = 60.0, *E*_*S*‖_ = 0.0 for (*a*) and −4.0 for (*b*), *v*_‖_ = 0.9806, *v*_?_ = 0.1961, *v*_*ph*_ = 1.101, *k*_*E*_ = 5.0, *k*_*B*_ = 10.0. All of the parameters are normalized as in ref. 18.

The theoretical curve in Fig. 5(b) are obtained with *v*_‖_ = 0.99*c* and *n*_*e*_ = 0.5*n*_*c*_. With 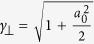 for linearly polarized laser pulses, Eq. (6) is modified as 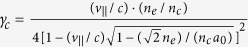.

## Additional Information

**How to cite this article**: Hu, R. *et al.* Dense Helical Electron Bunch Generation in Near-Critical Density Plasmas with Ultrarelativistic Laser Intensities. *Sci. Rep.* **5**, 15499; doi: 10.1038/srep15499 (2015).
